# Induced smectic phase in binary mixtures of twist-bend nematogens

**DOI:** 10.3762/bjnano.9.122

**Published:** 2018-04-26

**Authors:** Anamarija Knežević, Irena Dokli, Marin Sapunar, Suzana Šegota, Ute Baumeister, Andreja Lesac

**Affiliations:** 1 Division of Organic Chemistry and Biochemistry, Ruđer Bošković Institute, Bijenička cesta 54, 10000 Zagreb, Croatia; 2 Division of Physical Chemistry, Ruđer Bošković Institute, Bijenička cesta 54, 10000 Zagreb, Croatia; 3 Institute of Chemistry, Physical Chemistry, Martin Luther University Halle-Wittenberg, von-Danckelmann-Platz 4, 06120 Halle, Germany

**Keywords:** binary mixture, liquid crystals, smectic phase induction, temperature-dependent FTIR, twist-bend nematogen

## Abstract

The investigation of liquid crystal (LC) mixtures is of great interest in tailoring material properties for specific applications. The recent discovery of the twist-bend nematic phase (N_TB_) has sparked great interest in the scientific community, not only from a fundamental viewpoint, but also due to its potential for innovative applications. Here we report on the unexpected phase behaviour of a binary mixture of twist-bend nematogens. A binary phase diagram for mixtures of imino-linked cyanobiphenyl (CBI) dimer and imino-linked benzoyloxy-benzylidene (BB) dimer shows two distinct domains. While mixtures containing less than 35 mol % of BB possess a wide temperature range twist-bend nematic phase, the mixtures containing 55–80 mol % of BB exhibit a smectic phase despite that both pure compounds display a Iso–N–N_TB_–Cr phase sequence. The phase diagram shows that the addition of BB of up to 30 mol % significantly extends the temperature range of the N_TB_ phase, maintaining the temperature range of the nematic phase. The periodicity, obtained by atomic force microscopy (AFM) imaging, is in the range of 6–7 nm. The induction of the smectic phase in the mixtures containing 55–80 mol % of BB was confirmed using polarising optical microscopy (POM), differential scanning calorimetry (DSC) and X-ray diffraction. The origin of the intercalated smectic phase was unravelled by combined spectroscopic and computational methods and can be traced to conformational disorder of the terminal chains. These results show the importance of understanding the phase behaviour of binary mixtures, not only in targeting a wide temperature range but also in controlling the self-organizing processes.

## Introduction

Nowadays liquid crystal (LC) substances possess a wide range of uses. However, it is rather rare that a single organic compound has the desired properties for a particular application. Since the discovery that mixtures of nematic compounds could yield room-temperature nematic liquid crystals [1], the mixing of LC compounds became a very useful technique. Thus, investigation of LC mixtures is of great interest in targeting a wide operating temperature range or tailoring material properties for specific applications.

The recent discovery of the twist-bend nematic phase (N_TB_) [2–3] has sparked a great interest in the scientific community, not only from the fundamental viewpoint but also due to its potential for innovative applications. Even before this new nematic phase was described as a twist-bend nematic phase, the mixtures of LC compounds exhibiting it were prepared and investigated. The aim of those studies was to further explore this unknown nematic phase [4–6] or to confirm which new dimers exhibit this phase [7–9]. Consequently, investigations of mixtures containing twist-bend nematogens became of great interest since the N_TB_ phase can be induced and stabilized by the addition of a methylene-linked dimer possessing this phase to an ether-linked dimer which does not exhibit a N_TB_ phase [10–12].

Apart from shifting the phase transition temperatures in the mixtures of two LC compounds, the formation of a new mesophase is also an interesting phenomenon. The first induced mesophase was discovered in binary rod-like nematic liquid crystal mixtures of *N*-(*p*-methoxybenzylidene)-*p*-*n*-butylaniline (MBBA) with 4-cyano-4’-pentylbiphenyl (5CB) [13]. It has been associated with the formation of charge transfer (CT) complexes between strong donor and acceptor compounds. However, the induction of a smectic phase may also be the result of weak CT interactions, together with other effects such as dipole–dipole interactions, dipole-induced dipole interactions, and excluded volume effects [14–15].

**Figure 1:**
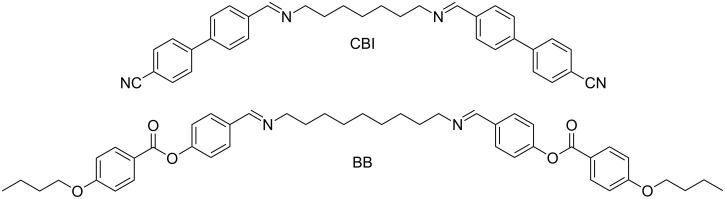
Molecular structures of cyanobiphenyl dimer (CBI) and benzoyloxy-benzylidene dimer (BB).

In this study, we report on the unusual behaviour of a binary mixture between two twist-bend nematogens. Previously we reported on the mesomorphic behaviour of various imino-linked dimers that displayed both uniaxial nematic (N) and twist-bend nematic phases (N_TB_) [16–17]. Continuing our investigation on these systems, we performed a study on how mixing two imino-linked bent-shaped dimers with a rather large difference in molecular length affects their mesomorphic properties. For the purpose of this study, we prepared an imino-linked cyanobiphenyl dimer (CBI**)** and an imino-linked benzoyloxy-benzylidene dimer (BB) having molecular lengths (in the most extended form) of 3.9 nm and 4.8 nm, respectively. The molecular structures of these compounds are presented in Figure 1. For both compounds, the Iso–N–N_TB_ phase sequence have been reported [16,18]. Although both pure constituents display only the nematic phases, the mixtures enriched with BB show an additional intercalated smectic phase. To our knowledge, this is the first case of an induced smectic phase in the mixture of two nematogens that exhibit the N_TB_ phase. Various spectroscopic techniques and molecular dynamic calculations were used in an attempt to determine the interactions responsible for the induction of the smectic phase. Atomic force microscopy (AFM) measurements performed on the mixtures enriched with CBI showed that the distance between modulations in N_TB_ phase is extremely short, just about 6–7 nm.

## Results and Discussion

Mesogenic properties of the pure compounds CBI and BB are presented in Table 1. The transition temperatures are determined by differential scanning calorimetry (DSC) and correspond to the data reported previously [16,18]. The N–N_TB_ phase transition has been confirmed observing characteristic blocky texture from which polygon and rope textures developed. The X-ray diffraction (XRD) measurements in both nematic phases show that the *d* value for the maxima of the inner scattering in the nematic phase is at about 1.4 nm for CBI and at about 2.2 nm for BB, which is less than the half the molecular lengths.

**Table 1:** The phase behaviour and molecular length, *L*, obtained at the B3LYP/6-31G level of density functional theory (DFT). The phase transition temperature (°C) and Δ*S*/*R* (given in brackets, dimensionless quantity) are summarized.

Dimer							*L* (nm)

CBI	Cr	117 [10.54]	N_TB_ 123 [0.01]	N	149 [0.14]	Iso	3.9
BB^a^	Cr	97 [6.86]	(N_TB_ 95) [0.12]	N	117 [0.19]	Iso	4.8

^a^Cr–Cr transitions at 86 °C [6.02] and at 91 °C [1.83]; value in parenthesis is the monotropic phase transition.

**Figure 2:**
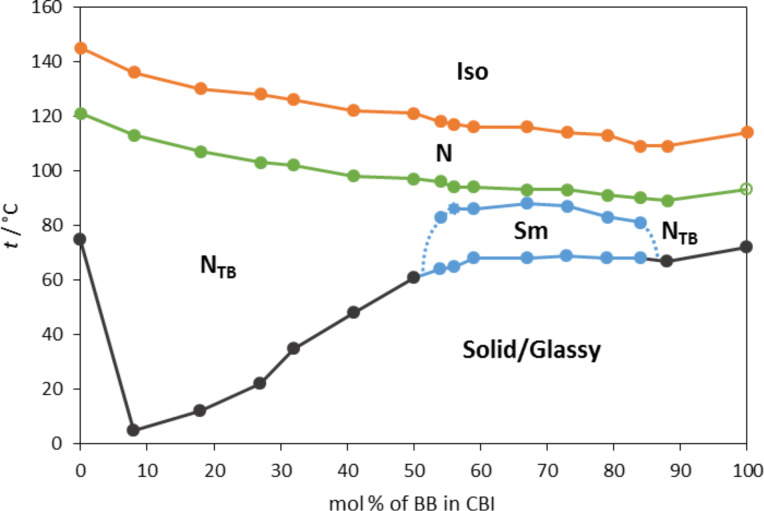
Phase diagram for binary mixture of CBI and BB.

The phase behaviour of binary mixtures of CBI and BB is characterized as a function of mol percent of BB and shown in Figure 2. To obtain the values under comparable conditions, the transition temperatures of the pure compounds and their mixtures were taken from the second DSC cooling run. This resulted in a slight lowering of the transition temperatures for the pure compounds comparable to the data obtained during the heating cycle (Table 1). The nature of the phase in various mixtures was identified by its characteristic texture. As can be seen in Figure 2, upon addition of BB to CBI, the Iso–N, as well as the N–N_TB_ transition temperatures, decrease linearly with increasing BB content. In terms of a molecular field theory developed to predict phase diagrams for binary mixtures of nematics [19], this suggests that the anisotropic intermolecular energy parameter between the unlike species is the geometric mean of the anisotropic interaction parameters between the like species. The linearity of both transition temperatures was also observed in the mixtures of methylene and ether-linked dimers and used for determination of the virtual nematic to N_TB_ transition temperatures of the later [20].

For the crystallization points, two trends are detectable. Upon addition of 8 mol % of BB to CBI, the transition to the solid phase decreases to 3 °C, compared to 75 °C of the pure CBI. Increasing the concentration of BB further up to 50 mol % causes a rather steep temperature increase up to 60 °C. In the range of 56 < mol % BB < 88 crystallization temperatures remain approximately the same at about 68 °C.

Considering the phase behaviour of the pure compounds, the expected Iso–N–N_TB_ phase sequence was observed across the full composition range between CBI and BB, however the most surprising was detection of the smectic phase for the mixtures containing 55–80 mol % of BB. Thus the phase diagram of the binary BB–CBI mixture can be envisaged as two distinct puzzling domains, one enriched with CBI and the other enriched with BB.

**Figure 3:**
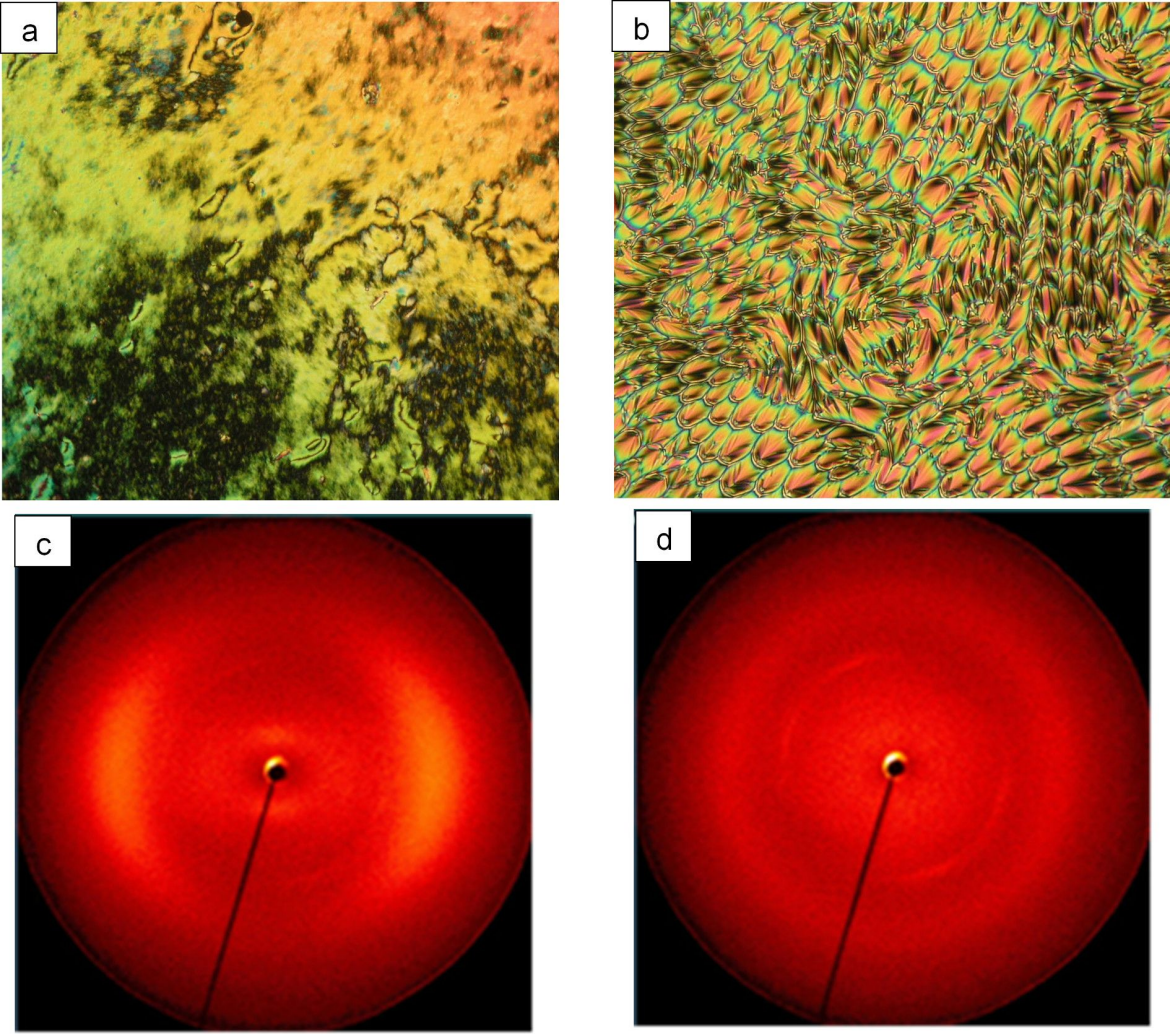
a) Marble texture of the nematic phase (magnification 200×); b) polygonal texture of the N_TB_ phase of 18 mol % BB mixture at 92 °C (magnification 500×), c) 2D XRD pattern in the N phase at 120 °C, d) 2D XRD pattern in the N_TB_ phase at 55 °C.

On cooling from the isotropic liquid, mixtures containing less than 50 mol % of BB show the nematic and the N_TB_ phase identified by marble (Figure 3a) and polygonal texture (Figure 3b), respectively. Further cooling resulted in increased viscosity and vitrification with no change in texture.

The assignment of the N_TB_ phase was further supported by small-angle X-ray diffraction studies on the mixture containing 18 mol % BB as shown in Figure 3. X-ray diffraction measurements of the sample were performed under a magnetic field of 1 T upon cooling (1 K/min) from the isotropic liquid. A typical diffuse small-angle scattering pattern was obtained for the nematic phase (Figure 3c). The pattern of the N_TB_ phase differs from that of the N phase mainly by a certain loss of orientation (Figure 3d and Figure S1, Supporting Information File 1 for additional data) as frequently found at the N–N_TB_ transition [2,17,21]. The inner scattering is too weak to find the maximum but can be estimated to be near that of the N phase, which has its maxima at about 1.8 nm for the *d* value.

It has been reported that freeze fracture TEM [22–25] and AFM [26–27] measurements of the N_TB_ phase show periodic features that can be related to a nanometer-scale pitch. Up to 40 mol % of BB large suppression of crystallization resulted in a wide temperature range of the N_TB_ phase. This enabled direct comparison of the surface morphology and periodicity modulations between several mixtures of different concentration of BB by atomic force microscope (AFM). Three mixtures containing 8, 18 and 27 mol % BB were investigated by AFM at 55 °C. For all three mixtures, the presence of the N_TB_ phase at 55 °C, was confirmed by characteristic texture measurements and additionally verified by the XRD measurement performed on the mixture containing 18 mol % of BB.

**Figure 4:**
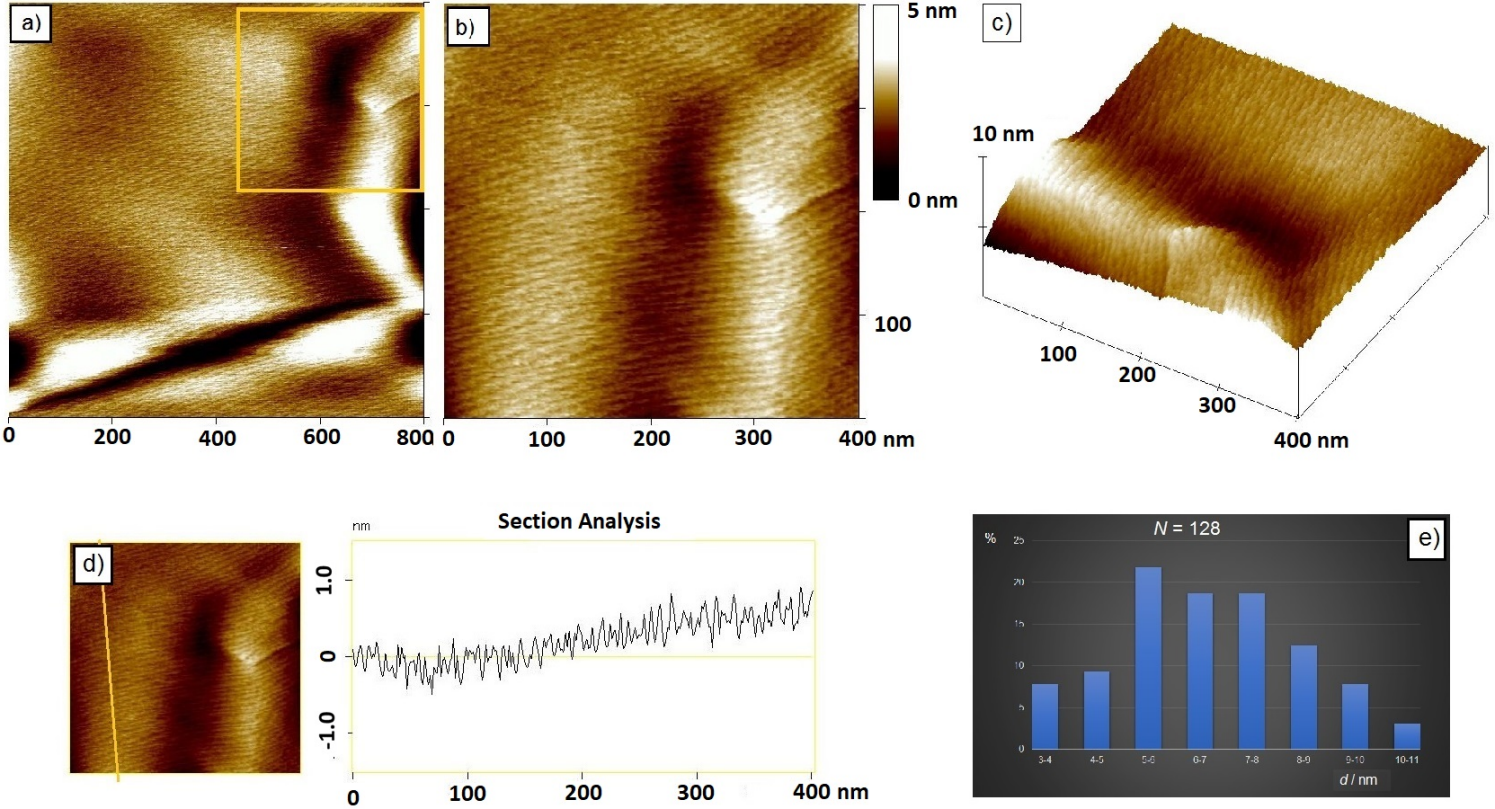
a) AFM 2D-topographic image of the surface morphology of a binary mixture of 27 mol % BB; b) the inset shows a high-resolution AFM image of the marked region in a); c) 3D-topographic image; d) vertical section profile across the noted line; e) histogram of the fingerprint modulation distance.

AFM imaging revealed very similar surface morphology for all three mixtures. The representative AFM images obtained on the mixture containing 27 mol % BB at 55 °C are shown in Figure 4.

In the N_TB_ phase, at 55 °C, a relatively smooth surface over 1 × 1 µm^2^ followed by the semi-circular shape as a part of toric domains was observed (Figure 4a). The boundaries of domains are characterized by the slight decrease in the surface height followed by the slight increase in height (Figure 4c). Within observed domains, a well-oriented periodic pattern appeared (Figure 4b). The distance between the fingerprint modulations determined by the cross-section analysis revealed that all three mixtures possess approximately the same periodicity amounting to 6.4 ± 1.7 nm (Figure 4d). The corresponding histogram of the distance between the fingerprint modulations is shown on the Figure 4e.

**Figure 5:**
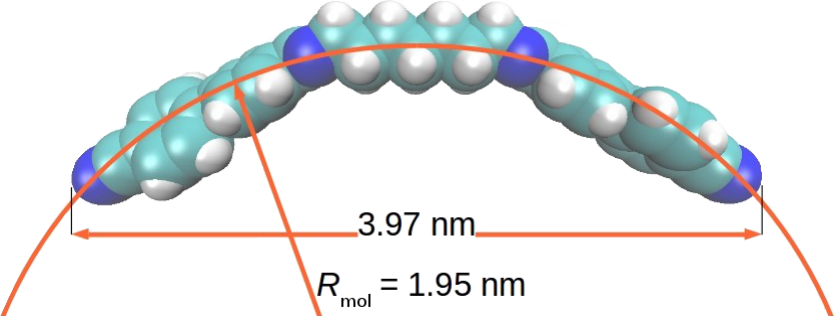
Molecular curvature of CBI obtained by a least squares fit of a circle to the geometry of the molecule.

Most recently Clark’s group indicated that the helix pitch (*p*_H_) of CB7CB experimentally measured by resonant soft X-ray scattering (RSoXS) appears to be controlled by the molecular bend and can be approximated with 2π*R*_mol_ of a single all-trans molecule near the N–N_TB_ phase transition [24]. For the CB7CB molecule, *R*_mol_ was determined to be 1.58 nm which gives *p*_Hlim_ ≈ 9.8 nm. Also, it was demonstrated that a small concentration of 5CB in the CB7CB–5CB mixtures has a negligible effect on the pitch. Compared to CB7CB, the CBI molecule is longer by two additional imino linkage groups. Investigation of the conformational distribution of the achiral symmetric dimers in the nematic and N_TB_ phases revealed high probability of more elongated conformations in the liquid crystalline phases [28–30]. Similar to CB7CB, the most elongated conformer of CBI is the one possessing alkyl spacer in all-trans conformation. The determined *R*_mol_ for CBI is higher than for CB7CB and equals 1.95 nm (Figure 5). Consequently, the predicted pitch is approximately 12.2 nm. The periodicity we obtained by AFM is in the range of 6–7 nm, which is approximatively half of the predicted pitch value. Considering the duplex helical tiled chain (DHTC) model proposed by Clark’s group [24], periodicity observed by AFM may be interpreted as biaxiality pitch caused by variations of the steric shape along the duplex chain.

**Figure 6:**
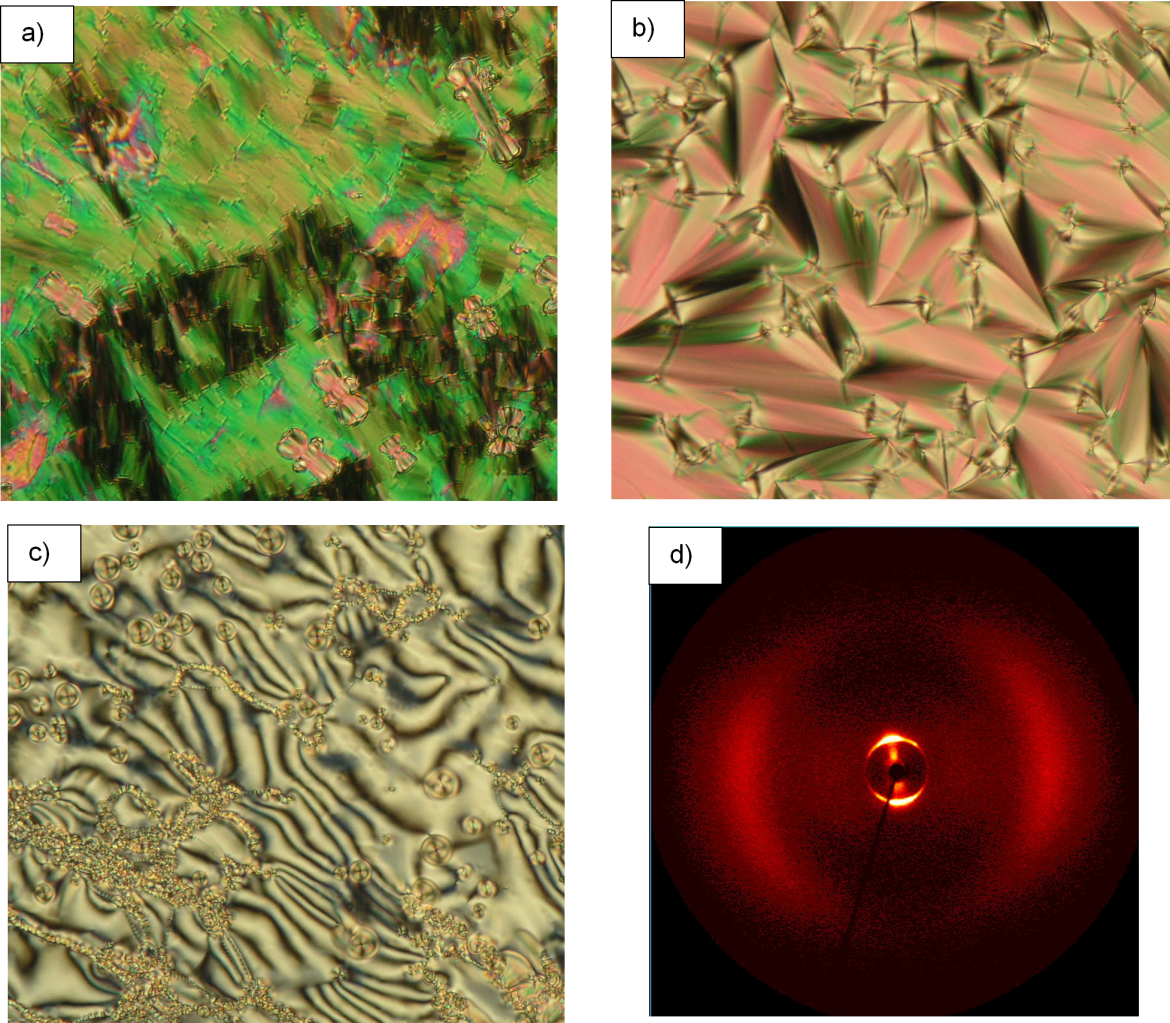
Texture of the mixture containing 73 mol % BB: a) blocky texture of N_TB_ phase at 93 °C, b) fan-shaped texture of the smectic phase at 82 °C, c) schlieren texture after shearing the sample at 82 °C, d) 2D XRD patterns for a sample of 73 mol % BB mixture aligned in the magnetic field obtained on cooling from the isotropic liquid at 73 °C.

Upon increasing the concentration of BB, the phase sequence changed. The mixtures containing 55–80 mol % of BB along with N–N_TB_ phase transition also show N_TB_–Sm phase transition. Upon cooling from the nematic phase, a blocky texture (Figure 6a) of the narrow N_TB_ phase appears, followed by a fan-shaped texture (Figure 6b) of the smectic phase. Shearing the sample led to a schlieren-like texture with singularities of *S* = ±1 and ±1/2 (Figure 6c) which have also been observed in anticlinic smectic C (SmC_A_) of a limited number of dimers displaying the same phase sequence [3,31–33]. The occurrence of such singularities is attributed to an opposite tilt direction of the mesogenic groups between adjacent layers [34]. The induction of the smectic phase was confirmed by the presence of an additional peak in the DSC trace and by X-ray diffraction analysis performed on the mixture containing 73 mol % of BB. The diffraction pattern obtained in magnetic field of 1 T upon cooling at 73 °C shows a broad diffuse outer scattering and a sharp layer reflection at *d* = 2.05 nm, the maxima of the inner and outer scattering remain on the meridian and the equator, respectively, but with a comparatively broad azimuthal distribution of the intensity (Figure 6d and Figure S2, Supporting Information File 1). This is in line with an intercalated smectic C phase. Given that the mesophase exhibits a schlieren texture with both 2- and 4-brush defects it is concluded that the phase is an anticlinic smectic C (SmC_A_) type. The induced smectic phase is most stable in the mixture containing roughly 70 mol % of BB. This corresponds to the ratio of two molecules of BB versus one molecule of CBI in the smectic phase. The entropy changes (Δ*S*/*R*) determined from the DSC thermograms (Table S1, Supporting Information File 1) for the N_TB_ to SmC_A_ transition exhibit the maximum around 70 mol % of BB which correspond to the highest N_TB_ to SmC_A_ transition temperature. It is also significantly larger than for the N–N_TB_ transition (0.56 versus 0.09) points that N_TB_ to SmC_A_ transition is first order.

The induced smectic phase can be compared with intercalated smectic phases observed for non-symmetric dimers. On entropic grounds, the intercalated arrangement in which there is a random mixing of the two different types of mesogenic units is favourable. The driving force for the formation of this phase was attributed to a specific interaction between the unlike mesogenic units [35–36].

In an attempt to determine the interactions responsible for induction of smectic phase UV, and IR measurements were performed on pure compounds and 73 mol % BB mixture. UV measurements were performed at room temperature in ethanol. Comparison of the UV spectra of the mixtures (18 mol % BB, 50 mol % BB, 67 mol % BB) with those of pure compounds shows no new absorption band (Figure S3, Supporting Information File 1). The peak at 290 nm present in pure CBI shifts toward lower wavelengths as the ratio of BB in the mixture rises, approaching the value of 266 nm characteristic of pure BB.

**Figure 7:**
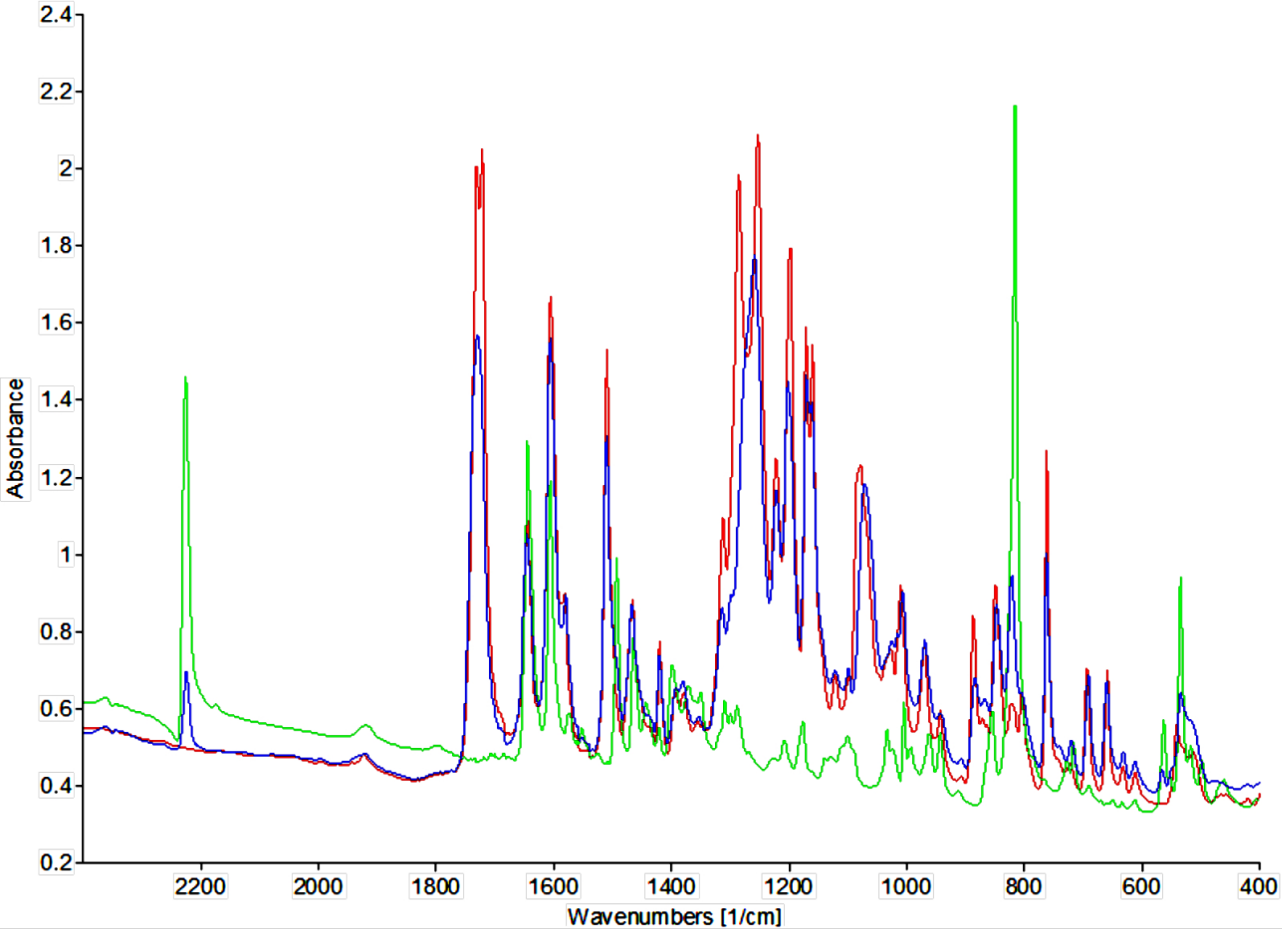
IR spectra of pure BB (red), pure CBI (green) and a 73 mol % BB mixture (blue) at room temperature.

Since FTIR spectroscopy is a sensitive technique for determining changes in chemical interactions and molecular geometry in LC phases [37–40], we investigated the 73 mol % BB mixture using temperature-dependent FTIR measurements. IR spectra were recorded using a KBr pastille method. Since in a binary mixture the cyano group of CBI may be involved in interaction with BB in the mixture, we focused our attention on the IR band related to the stretching of the C≡N bond. Comparison of the spectra of pure compounds and 73 mol % BB mixture at room temperature reveals that shift of C≡N stretching vibration is negligible (Figure 7). The observed difference is a band shift of 816 cm^−1^ for pure CBI to 822 cm^−1^ for the 73 mol % BB mixture. This band corresponds to the out-of-plane bending of the aromatic ring. Also, the difference in the spectra of pure BB and 73 mol % BB mixture is a shift of band at 1080 cm^−1^ for pure BB to 1072 cm^−1^ for mixtures and a significant decrease of the band at 1286 cm^−1^ compared to pure BB. These bands correspond to stretching vibrations of C–O–C bonds. Similar results were obtained after the analysis of the 73 mol % mixture spectra recorded during the cooling cycle from isotropic phase (Figure S4, Supporting Information File 1) and temperature-dependent spectra of pure BB (Figure S5, Supporting Information File 1).

The absence of new absorption bands in the wide spectral range in the mixture, compared to the spectra of the pure compounds, implicates that CT attraction or even dipole-induced dipole interaction is not particularly strong. In the absence of electronic arguments, we envisaged other intermolecular interactions that might promote smectic arrangement.

**Figure 8:**
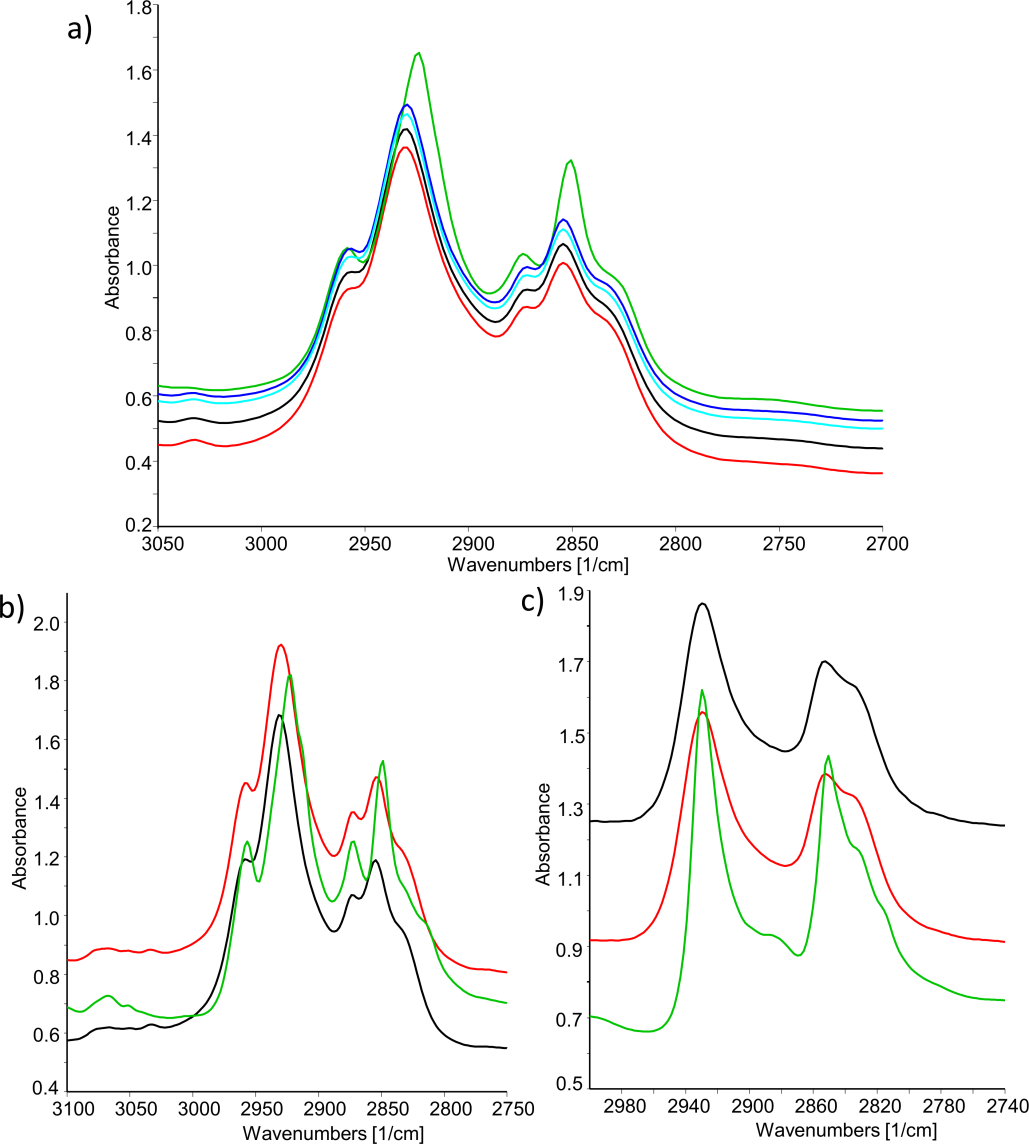
IR spectra in the CH_2_ stretching bands region of: a) 73 mol % mixture – 50 °C (green), 75 °C (blue), 90 °C (cyan), 100 °C (red), 125 °C (black), b) pure BB – 25 °C (green), 95 °C (black), 125 °C (red), c) pure CBI – 25 °C (green), 130 °C (black), 150 °C (red).

Further analysis of the spectra of the mixture show that the bands of the symmetric and asymmetric stretching vibrations of CH_2_ units (ν_s_(C−H)CH_2_ and ν_as_(C−H)CH_2_) at 2924 cm^−1^ and 2850 cm^−1^ are shifted to 2930 cm^−1^ and 2854 cm^−1^ upon heating and restore to their values upon cooling (Figure 8a). The change occurs at the transition from crystal to smectic phase and the values remain the same in LC and isotropic phases. This well-known shift in the IR spectra of liquid crystals is a consequence of disordering of chain packing and the introduction of gauche conformers on the alkyl chains which leads to their more liquid-like state [38,41–43]. This change is most evident at the crystal–smectic transition since in the solid state the chains are considered to be mostly trans-planar [44]. The increase in hydrocarbon chain conformational disorder has also been intensively studied by FTIR on phospholipid bilayers and is used for monitoring of lipid hydrocarbon chain melting phase transitions [39,45–47]. The temperature dependent IR spectra of pure BB (Figure 8b) follow the same trend as the spectra of 73 mol % mixture. The bands of CH_2_ vibrations shift toward higher frequencies. The spectra of CBI show different characteristics (Figure 8c). There is no shift of corresponding bands upon heating and only the bandwidth increases, which is in accordance with the increase of the motional rate of the molecule [37,39,41]. Considering the chemical structure of both pure compounds, it is reasonable to assume that shifting of CH_2_ vibrations rises from conformational disorder of terminal chains present only in the structure of BB.

**Figure 9:**
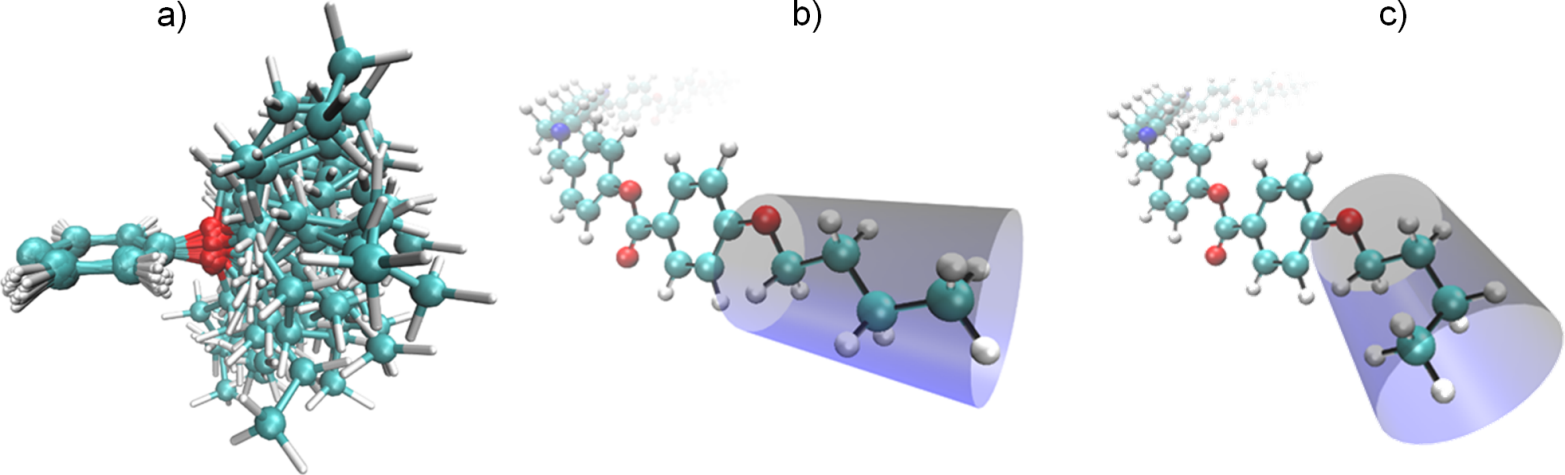
a) Free rotation of the C–C bonds of the alkyl chains in the gas phase molecular dynamics. Snapshots were taken every 0.2 ps from a 10 ps molecular dynamics simulation at 80 K. To illustrate the motion of the alkyl chain separately from the motion of the remainder of the molecule, the snapshots are shown with the benzene ring of each geometry aligned, and only the alkyl chain and benzene ring are shown. b) All-trans conformation and c) single gauche conformations of the alkyl chain of BB. The volume excluded by the alkyl chains is marked by the blue cylinders.

In order to investigate how conformational disorder of terminal chains might affect induction of the smectic phase we performed a molecular dynamics simulation of BB at 80 K with a time step of 1 fs for a total duration of 10 ps using the Turbomole program package [48] at the PBE/def2-TZVP level of theory using the RI approximation [49]. The dynamics were performed in the molecular frame, the centre of mass of the molecule was kept at the origin of the coordinate system and the molecule was rotated to satisfy the Eckart conditions. The geometry of the entire molecule was varied during the simulation, and discrete conformers were sampled every 0.2 ps from the dynamics. The sampled geometries are shown in Figure 9a. The snapshots are aligned to maximize the overlap of the benzene ring of each geometry. In this way, the motion of the alkyl chain during the dynamics can be seen clearly. Both the all-trans conformer and various gauche conformers are present among the geometries, indicating that the transition between the conformers occurs freely at 80 K. In a crystal, steric effects favour the all-trans conformation where the chains occupy the lowest volume (Figure 9b). As the temperature increases, the probability for the gauche conformation in the chain also increases, which leads to a shortening of the chains but an increase in the volume they occupy by ≈10–15% (Figure 9c).

Generally, the intercalated structures are typical for non-symmetric dimers [50]. The driving force for the formation of this phase was attributed to a specific interaction between the unlike mesogenic units [34–36]. In binary mixtures, enhanced or induced smectic behaviour is most often associated with a concurrent upward curvature of the T_N-I_ line, and such behaviour is normally associated with a specific interaction between the unlike mesogenic groups [34]. For the BB–CBI mixtures both the Iso–N and the N–N_TB_ transition temperatures change linearly. Furthermore, the phase diagram of the binary mixtures of CB9CB and the benzyloxyphenyl-based dimer display only nematic phases, which is in accordance with the behaviour of pure compounds [51]. This data combined with the temperature-dependent FTIR measurements suggest that a specific interaction between the different mesogenic groups is not particularly strong. According to the phase diagram, the induced smectic phase is most stable in the mixture containing roughly 70 mol % of BB and we focused our attention on how can smaller CBI molecule facilitate the intercalated smectic phase of symmetric BB.

**Figure 10:**
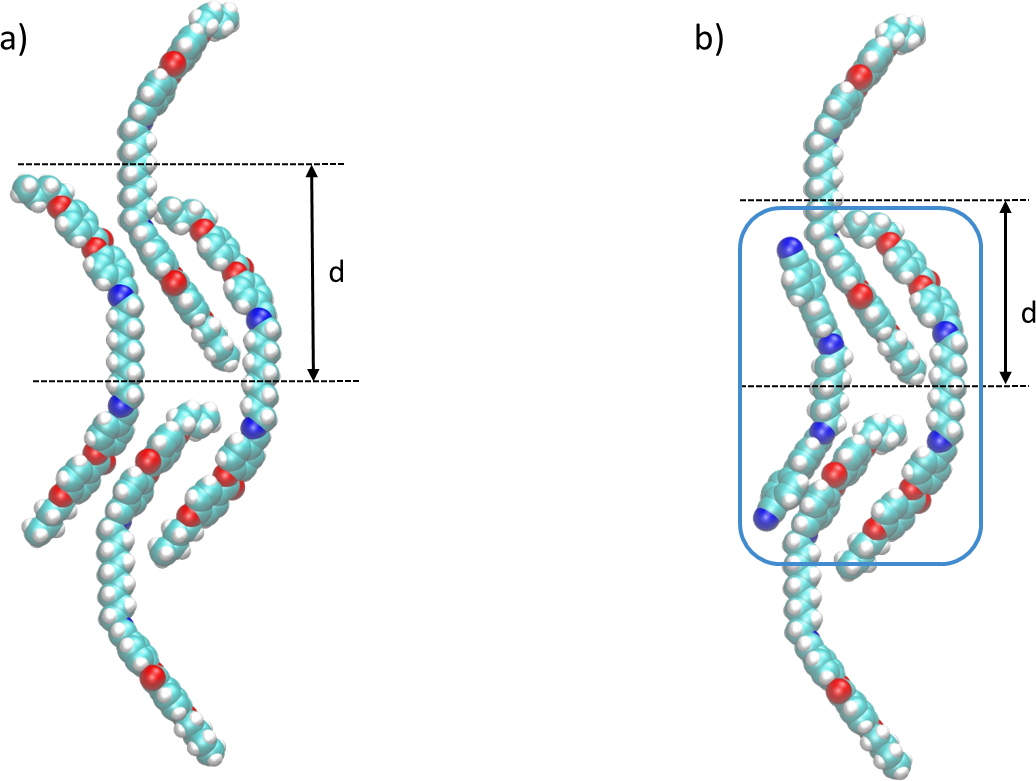
a) A sketch of the hypothetical intercalated smectic-like organization of pure BB, where *d* denotes layer spacing. b) A sketch of intercalated smectic phase of a BB–CBI mixture comprising the ratio of two molecules of BB versus one molecule of CBI (blue square).

It is well known that within the intercalated smectic phase dimeric molecules are arranged in the way in which terminal chains and the spacers are mixed randomly and the layer spacing is approximately half the molecular length [50]. As evidenced from the temperature-dependent FTIR measurements, part of the terminal chains adopt a gauche conformation and become shorter. Placing the molecules of BB in the hypothetical intercalated smectic-like arrangement generates void space near the spacer (Figure 10a). Since void space in molecular packing is unacceptable for condensed mesophase formation [52–53] it is reasonable to expect destabilization of the SmC_A_ phase. Indeed, only the Iso–N–N_TB_ phase sequence was observed for the pure BB. In the BB–CBI mixtures, introduction of smaller CBI molecules facilitate the space filling and stabilize packing within the smectic phase (Figure 10b). Thus, induction of the intercalated smectic phase in the BB–CBI mixtures enriched with BB can be attributed to the minimization of the free volume, although synergy with the weak electrostatic quadrupolar interaction between these particular mesogenic groups cannot be excluded.

## Conclusion

Here we report on the unexpected phase behaviour of a binary mixture of twist-bend nematogens. The phase diagram of the binary BB–CBI mixtures shows two distinct and very puzzling domains. While mixtures containing less than 35 mol % of BB possess a wide temperature range twist-bend nematic phase, the mixtures containing 55–80 mol % of BB exhibit a smectic phase despite that both pure compounds display a Iso–N–N_TB_–Cr phase sequence. The phase diagram shows that introduction of a significantly longer molecule suppresses crystallization, yielding a wide temperature range of the N_TB_ phase and maintaining the temperature range of the nematic phase. For the mixtures containing up to 30 mol % of BB, AFM surface analysis revealed approximately the same periodicity of 6–7 nm. This demonstrates the utility of making mixtures to obtain a wide temperature range N_TB_ phase. An increase in the concentration of BB resulted in the formation of the intercalated smectic phase. The induction of a smectic phase was confirmed using polarising optical microscopy (POM), differential scanning calorimetry (DSC) and X-ray diffraction. The temperature-dependent FTIR measurements revealed that the origin of the intercalated smectic is likely to be a conformational disorder of terminal chains instead of a charge transfer (CT) complex or dipole-induced dipole interaction. Comparison of the packing models of pure BB and CBI–BB mixtures enriched with BB strongly suggests that the SmC_A_ phase formation is driven by minimization of the free volume, although synergy with the weak electrostatic quadrupolar interaction between these particular mesogenic groups cannot be excluded. The above-presented results show that mixing two twist-bend nematogens not only results in the extended temperature range of the N_TB_ phase but also a phase sequence can be changed. Thus, targeting a wide operating temperature range or controlling the self-organizing processes makes understanding the phase behaviour of binary mixtures very important.

## Supporting Information

File 1 Additional experimental and spectroscopic information.
General information; phase transition temperatures for the mixtures of BB and CBI; UV spectra of pure BB and CBI and selected mixtures in ethanol; IR spectra of 73 mol % mixture in the region 1300–900 cm^−1^ at various temperatures; IR spectra of pure BB in the region 1800–1000 cm^−1^ at various temperatures.
