# Supplementary material for: Induced smectic phase in binary mixtures of twist-bend nematogens
Source: Beilstein J Nanotechnol. 2018 Apr 26;9:1297–307. doi: 10.3762/bjnano.9.122 (PMC5942249; doi:10.3762/bjnano.9.122)
Supplement: File 1 — Additional experimental and spectroscopic information. General information; phase transition temperatures for the mixtures of BB and CBI; UV spectra of pure BB and CBI and selected mixtures in ethanol; IR spectra of 73 mol % mixture in the region 1300–900 cm−1 at various temperatures; IR spectra of pure BB in the region 1800–1000 cm−1 at various temperatures. [file Beilstein_J_Nanotechnol-09-1297-s001.pdf]

Supporting Information

for

Induced smectic phase in binary mixture of twist-bend nematogens

Anamarija Knežević[1], Irena Dokli[1], Marin Sapunar[2], Suzana Šegota[2], Ute Baumeister[3] and Andreja Lesac*[1]

Address: [1]Division of Organic Chemistry and Biochemistry, Ruđer Bošković Institute, Bijenička cesta 54, 10000 Zagreb, Croatia, [2]Division of Physical Chemistry, Ruđer Bošković Institute, Bijenička cesta 54, 10000 Zagreb, Croatia and [3]Institute of Chemistry, Physical Chemistry, Martin Luther University Halle-Wittenberg, von-Danckelmann-Platz 4, 06120 Halle, Germany.

*Corresponding author

Email: Andreja Lesac - Andreja.Lesac@irb.hr

Additional experimental and spectroscopic information

General Information

Phase transition temperatures and textures were determined using an Olympus BX51 polarizing microscope equipped with a Linkam TH600 hot stage and a PR600 temperature controller. Enthalpies of transition were determined from thermograms recorded on a Perkin-Elmer DSC differential scanning calorimeter operated at scanning rates of 5 °C min^{-1}. Powder

X-ray patterns were obtained with a Guinier film camera (HUBER Diffraktionstechnik, Germany) using quartz-monochromated CuKa radiation and, for the small-angle region, with a modified Kratky camera and a one-dimensional position-sensitive detector (M. Braun, Germany) using Ni-filtered CuKa radiation from samples in glass capillaries (diameter 1 mm) mounted in temperature-controlled heating stages. Patterns of aligned samples (magnetic field of 1 T) on a glass plate on a temperature-controlled heating stage (alignment at the sample/glass or at the sample/air interface, sample fibre-like disordered around an ‘-fold axis perpendicular to this interface) were obtained with a two-dimensional detector (HI-STAR, Siemens). The atomic force microscopy (AFM) analysis of the surface of the sample was performed using a Multimode AFM with Nanoscope IIIa controller (Bruker Corporation, Billerica, MA, USA) with a vertical engagement (JV) 125 µm scanner. Tapping mode imaging was performed under ambient conditions in air, by using silicon tips (RTESP, Bruker, nom. freq. 320 kHz, nom. spring constant of 42 N/m) and at a scan resolution of 512 samples per line. The soft tapping mode imaging was performed at the set point ratio of 0.9 to avoid any destroying of the sample. The linear scanning rate was optimized to 1 Hz at a scan angle of 0°. The samples of binary mixtures on the glass plate are glued to the sample holder (stainless steel, diameter 1.5 mm) immediately before imaging. Imaging was performed with a temperature controller stage (Digital instruments, high temperature heater controller, range up to 60 °C, resolution 0.1 °C and accuracy 3%). Temperature setup consisted of a resistor placed between the scanner and the sample. It transmits heat to the sample from underneath. The whole sample remained attached to the microscope scanner by a magnet. Since the distance between the heating element and the sample itself is typically <2 mm, the temperature gradient between the displayed and the real temperature was insignificant. The temperature that the heater displays and the real temperature of the sample were the same. The imaging of samples was obtained at 55 °C. Processing and analysis of images were

carried out using the NanoScopeTM software (Digital Instruments, Version V531r1). Images were processed and analyzed by means of the AFM NanoScope software (version 5.12r5, Digital Instruments, Tonawanda, NY, USA). All images are presented as raw data except for the first-order two-dimensional flattening. Scanning rates were normally optimized around 1 Hz. The few sample surface areas were investigated to ensure that the scan areas of 1 μm × 1 μm or less were representative of the material features of interest. FTIR spectra were measured on an ABB Bomen MB102 spectrometer equipped with CsI 4 optics and DTGS detector. 73 mol % BB mixture, pure BB and pure CBI were recorded as pellets in KBr matrices in transmission mode with nominal resolution of 4 cm^{-1} (effective resolution 2 cm^{-1}) and 10 scans both at room temperature and in temperature-dependent regime. For the regulation of the heating rate, which was 5 K min^{-1}, Specac 3000 Series high stability temperature controller with heating jacket was used. Absorption IR spectra were recorded every 5 °C from room temperature to 150 °C ensuring the transition to isotropic phase of all samples. After accomplishing the temperature of 150 °C, the samples were cooled down at rate 5 °C min^{-1} to 50 °C. The samples were then cooled down to room temperature by standing on air for about one hour and prepared again as KBr pellet. The comparison of obtained IR spectrum and the first spectrum recorded at room temperature eliminated compound decomposition. The sample of the mixture was recorded during heating and cooling cycle.

Sample preparation for AFM

Samples for the AFM investigations were prepared as follows: Small amounts of the mixture were introduced into non-treated glass cell at the temperature above isotropization. Then the sample was cooled to 60 °C. The cells were disassembled by shifting the top glass plates. Then, the samples were annealed at 60 °C for 24 hours prior to AFM investigation.

Phase behaviour of binary mixture

Table S1: Phase transition temperatures [°C] of the mixture BB and CBI measured on cooling (5 °C/min).

BB/wt %	BB/mol %	Iso		N		N_{TB}		Sm		Solid
0	0	•	145	•	121	•			75	Cr
			[0.14]		[0.01]					
11	8	•	136	•	113	•			5	glassy
			[0.10]		[0.05]					
24	18	•	130	•	107	•			10	glassy
			[0.10]		[0.04]					
35	27	•	128	•	103	•			22	glassy
			[0.12]		[0.04]					
40	32	•	126	•	102	•			35	glassy
			[0.10]		[0.06]					
50	41	•	122	•	98	•			48	glassy
			[0.08]		[0.05]					
59	50	•	121	•	97	•			61	solid
			[0.10]		[0.06]					
62	54	•	118		96	•	83		64	solid
			[0.12]		[0.06]		[0.17]			
65	56	•	117	•	94	•	86	•	65	solid
			[0.12]		[0.06]		[0.18]			
67	59	•	116	•	94	•	86	•	68	solid
			[0.12]		[0.07]		[0.23]			
74	67	•	116	•	93	•	88	•	68	solid
			[0.11]		[0.07]		[0.55]			
79	73	•	114	•	93	•	87	•	69	solid
			[0.09]		[0.06]		[0.56]			
84	79	•	113	•	91	•	83	•	68	solid
			[0.10]		[0.05]		[0.29]			
88	84	•	109		80	•	81		68	solid
			[0.12]		[0.07]		[0.26]			
91	88	•	109	•	89	•			67	solid
			[0.15]		[0.07]					
100	100	•	108	•	88	•			72	Cr
			[0.19]		[0.12]					

XRD data

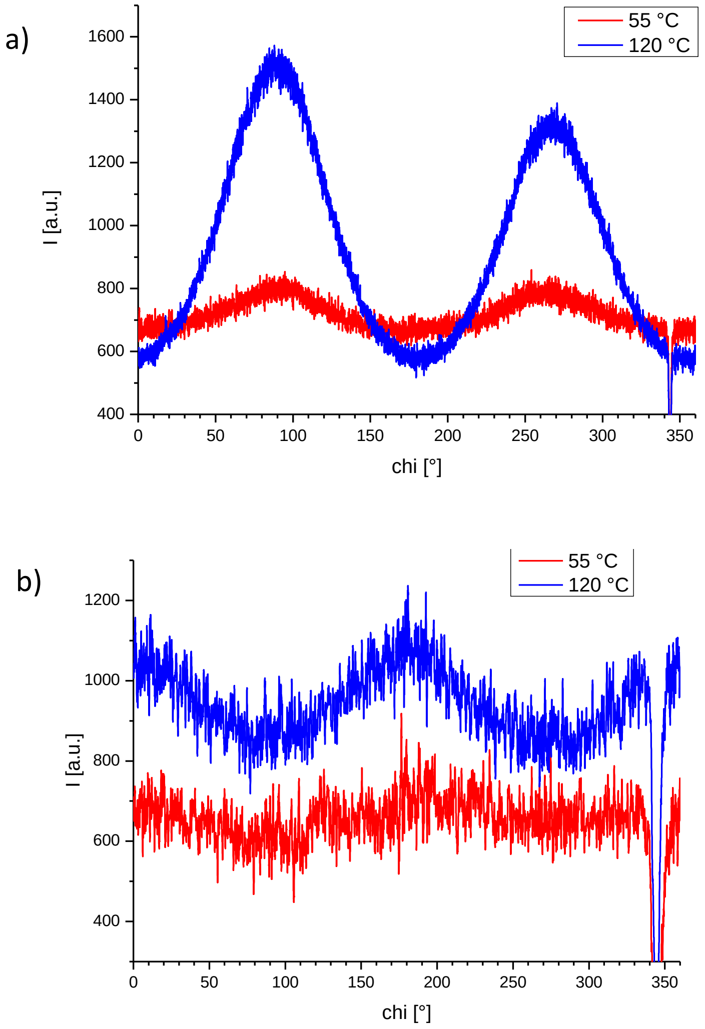

Figure S1: χ-scan for the mixture containing 18 mol % of BB aligned in a permanent magnetic field perpendicular to the incident beam and to the long axis of the capillary; a) χ-scan (2θ = 16–24°), outer diffuse scattering, b) χ-scan (2θ = 4–6°), inner diffuse scattering.

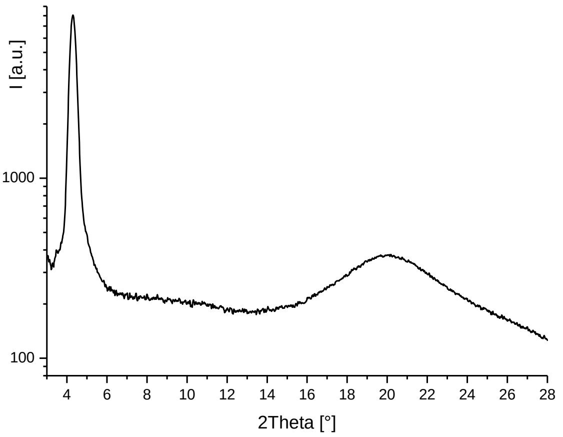

Figure S2: Theta-scans for the mixture containing 73 mol % BB aligned in a permanent magnetic field perpendicular to the incident beam and to the long axis of the capillary at 73 °C on cooling.

Spectroscopic data

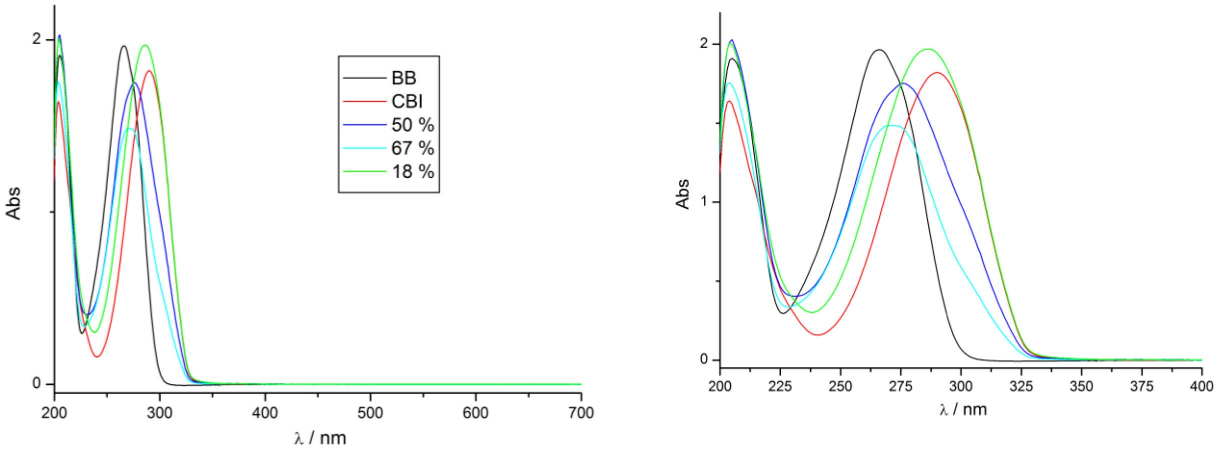

Figure S3: UV spectra of pure BB and CBI and prepared mixtures in ethanol at room temperature (maximum at 287 nm, 276 nm, 271 nm for 18 mol %, 50 mol % and 67 mol % respectively).

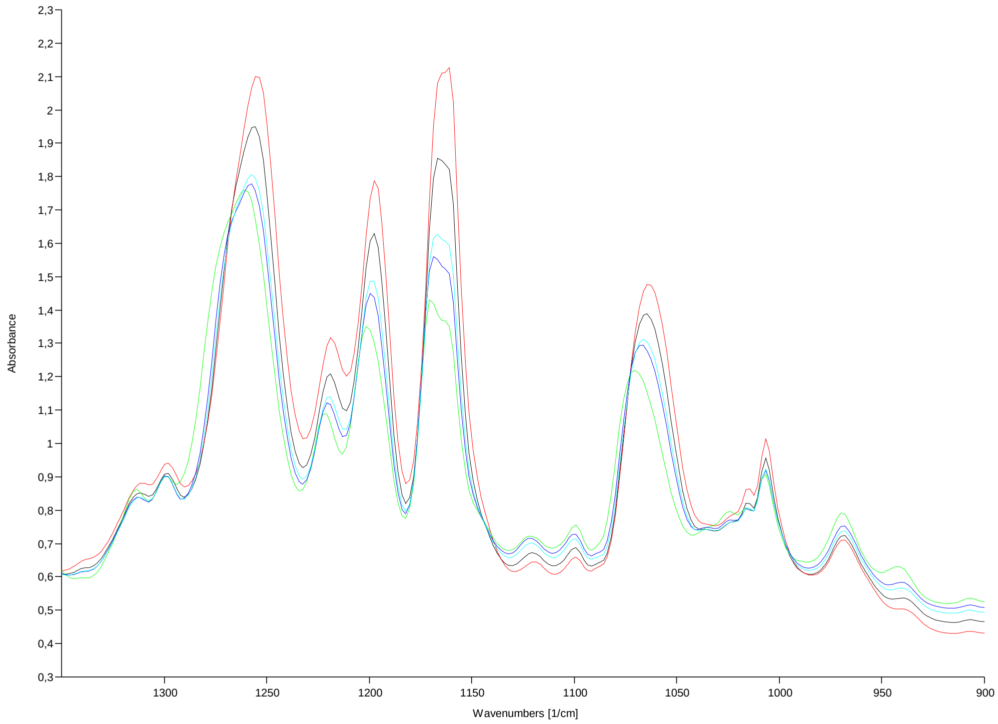

Figure S4: IR spectra of 73 mol % mixture in the region 1300–900 cm^{-1} at 50 °C (green), 75 °C (blue), 90 °C (cyan), 100 °C (black), 125 °C (red).

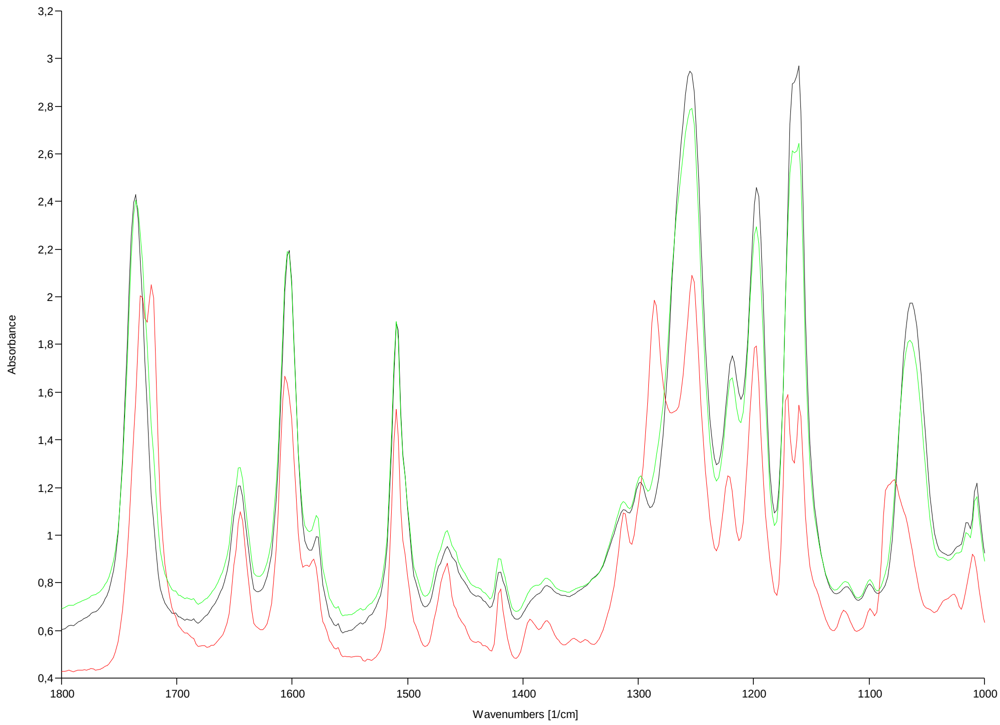

Figure S5: IR spectra of pure BB in the region 1800–1000 cm^{-1} at 25 °C (red), 95 °C (green), 125 °C (black).
